# The Impact of Dental Implant Surface Modifications on Osseointegration and Biofilm Formation

**DOI:** 10.3390/jcm10081641

**Published:** 2021-04-12

**Authors:** Stefanie Kligman, Zhi Ren, Chun-Hsi Chung, Michael Angelo Perillo, Yu-Cheng Chang, Hyun Koo, Zhong Zheng, Chenshuang Li

**Affiliations:** 1 School of Dental Medicine, University of Pennsylvania, Philadelphia, PA 19104, USA; sklig@dental.upenn.edu; 2 Biofilm Research Laboratories, Department of Orthodontics, Divisions of Pediatric Dentistry & Community Oral Health, School of Dental Medicine, University of Pennsylvania, Philadelphia, PA 19104, USA; zhiren@upenn.edu (Z.R.); Koohy@upenn.edu (H.K.); 3 Department of Orthodontics, School of Dental Medicine, University of Pennsylvania, Philadelphia, PA 19104, USA; chunc@upenn.edu (C.-H.C.); mperillo@upenn.edu (M.A.P.); 4 Department of Periodontics, School of Dental Medicine, University of Pennsylvania, Philadelphia, PA 19104, USA; yuchengc@upenn.edu; 5 Center for Innovation & Precision Dentistry, School of Dental Medicine and School of Engineering & Applied Sciences, University of Pennsylvania, Philadelphia, PA 19104, USA; 6 Division of Growth and Development, Section of Orthodontics, School of Dentistry, University of California, Los Angeles, Los Angeles, CA 90095, USA; 7 Department of Surgery, David Geffen School of Medicine, University of California, Los Angeles, Los Angeles, CA 90095, USA

**Keywords:** surface modification, dental implant, bone and soft tissue integration, titanium, osseointegration, biofilm

## Abstract

Implant surface design has evolved to meet oral rehabilitation challenges in both healthy and compromised bone. For example, to conquer the most common dental implant-related complications, peri-implantitis, and subsequent implant loss, implant surfaces have been modified to introduce desired properties to a dental implant and thus increase the implant success rate and expand their indications. Until now, a diversity of implant surface modifications, including different physical, chemical, and biological techniques, have been applied to a broad range of materials, such as titanium, zirconia, and polyether ether ketone, to achieve these goals. Ideal modifications enhance the interaction between the implant’s surface and its surrounding bone which will facilitate osseointegration while minimizing the bacterial colonization to reduce the risk of biofilm formation. This review article aims to comprehensively discuss currently available implant surface modifications commonly used in implantology in terms of their impact on osseointegration and biofilm formation, which is critical for clinicians to choose the most suitable materials to improve the success and survival of implantation.

## 1. Introduction

The history of dental implants dates as far back as 600 A.D. when the Mayan population used pieces of shells to replace missing teeth [1]. Fast forward to the 1930s, the first endosteal implant used in dentistry took after orthopedic screw fixtures and were made of Vitallium, a chromium-cobalt alloy [2]. To facilitate bone growth onto metal, a spiral stainless-steel implant was developed in the 1940s, which later evolved to a double-helical spiral implant. Remarkably, Dr. Per-Ingvar Brånemark introduced a threaded titanium root-form implant used on patients in 1965, and is the first stable dental implant to be well-documented [3]. Since then, implants have undergone a great evolution in shape, size, and surface to continuously improve implant survival and success. Simultaneously, specific criteria have been developed to objectively define the success of an implant, including immobility, absence of peri-implant radiolucency, marginal bone loss less than 1 mm during the first year in function and 0.2 mm annually after, a width of attached gingiva >2 mm, absence of pain, infection, paresthesia, other neuropathies, and the procedure performed without complications [3,4,5]. Unfortunately, of the five million implants placed by dentists in the United States per year, approximately 1–2% of patients experience primary implant failure due to inadequate osseointegration, while about 5% of patients experience secondary implant failure caused by peri-implantitis [6,7]. According to the criteria for implant success, an implant with these conditions is considered to be surviving rather than successful [8]. If these circumstances progress, immediate implant removal may be required. This is detrimental to the patient’s health, requiring added procedures, makes it more difficult to establish appropriate function and esthetics, and results in added medical costs. Moreover, replacing a previously failed implant in the same location has been shown to have survival rates as low as 71% since it is challenging to achieve osseointegration in a compromised bone site [8]. As the implant market continues to grow, with implant penetration in patients affected by tooth loss reaching 25–30% in the US in 2020, compared to 15–20% in 2011, ensuring implant success is crucial to mitigate a potential increased financial burden of failures [9].

In the 1950s, by placing titanium chambers into rabbit femurs, Dr. Per-Ingvar Brånemark pioneered the investigation to elucidate the biological relationship between implants and the surrounding bone [10]. His discovery of the process of osseointegration as “*a direct structural and functional connection between ordered, living bone, and the surface of a load carrying implant*” was a revolutionary concept in understanding the process of successful implant placement [11]. It is known that the first step toward osseointegration is primary stability, which is achieved at the time of surgical implant placement. As healing occurs and new bone appears, secondary stability is achieved [12]. Importantly, both stages of osseointegration can be influenced by implant characteristics. In particular, implant surface, which directly contacts the bio-environment, can greatly influence the biological response and impact the mechanical strength of the interaction between the implant and the tissue, playing a crucial role in determining the short- and long-term fate of the implant [13]. For instance, texturing increases surface area, which more effectively distributes stress, resulting in a more direct bone-to-implant contact. Further, surface properties that affect molecular interactions, cellular response, and bone regeneration can greatly determine the success of implantation [14]. In particular, those surfaces that can stimulate the growth of osteoblasts and/or their production of growth factors and cytokines will positively influence osseointegration. For example, by building up a nanoscale galvanic reduction−oxidation (redox) system on the metal implant surface, Zhang et al. introduced novel osteogenic bioactivity to the implant for promoting peri-implant bone growth [15].

No doubt, osseointegration is a competition between infectious organisms that seek to contaminate, colonize, and ultimately form biofilms on the implant surface versus the body’s own endogenous tissues that seek to grow onto the implants via osteogenesis. Thus, the most common complications related to implants initiate at the implant–bone interface [16]. Peri-implantitis is a biofilm-related, immune-mediated chronic inflammation affecting the implant sites and is characterized by loss of implant supporting bone [17]. Similar to the biofilm formation on natural teeth, bacterial colonization occurs within minutes after the implantation procedure and throughout the life cycle of an implant [18]. The accumulation of the biofilm and specific anaerobic pathogen are known as the primary etiology for the bone loss [19]. For over a decade, peri-implantitis was treated with mechanical debridement and antimicrobials that were used to treat periodontitis, a gum disease of natural teeth that appeared to have similar phenotypes including biofilm accumulation, signs of soft tissue inflammation, increased depth/bleeding on probing of the gingival pocket area, and destruction of the supporting bones [20,21]. However, several unsuccessful adaptions of periodontitis treatment strategies to peri-implantitis reveal the need to distinguish the peri-implantitis pathogens from the ones associated with periodontitis [22]. The lack of keratinized mucosa barrier around implants versus natural teeth was found to be associated with higher bacterial invasion and plaque accumulation [23]. Meanwhile, the pathogenesis of inflammatory destruction around an implant also differs from that around natural teeth in that the rate of bone degradation around an implant is more rampant and extensive—a substantial bone loss can be seen as early as six months post-implant placement [24]. Although both diseases are associated with a polymicrobial subgingival community and its dysbiosis, recent revisits on the disease’s microbiome indicated a generally higher prevalence of *Actinobacillus actinomycetemcomitans*, *Prevotella intermedia*, and *Tannerella forsythia* in peri-implantitis biofilms as compared to either periodontitis biofilms or healthy implants [25]. In addition, *Tannerella* spp., *Parvimonas* spp., *Fusobacterium* spp., and *Campylobacter* spp. are all among the frequently detected genera in peri-implant lesions [25], indicating that peri-implantitis is driven by a highly complex community through polymicrobial synergy and dysbiosis rather than individual causative pathogens, yet the characteristic peri-implantitis-related microbiome is not fully elucidated. Another over-looked risk factor of peri-implantitis is the abiotic surface which appears to alter the microbiota as the pathogenic biofilm develops [26,27], and release ions/particles as a result of the implant surface degradation triggering pro-inflammatory immune responses [28]. Due to the multifactorial nature of peri-implantitis biofilms and the risks associated with the abiotic surface, eradication becomes extremely challenging once a mature biofilm develops on an implant surface, even using antibiotic therapies and surgical irrigation or debridement [29,30]. Therefore, it is crucial for implant survival that surface materials could prevent primary adhesion of microbial cells by either repelling or killing the approaching bacteria. In particular, they must be prepared to combat pathogens most associated with the dental implant infections as mentioned above [31]. The need for accelerating osseointegration for rapid loading or at the compromised bone at the recipient site and for mitigating biofilm formation to decrease the occurrence of peri-implantitis has encouraged great advancements in implant surface modification [32]. Generally, patients can greatly benefit from these surface modifications that can promote osseointegration and mitigate biofilm formation to enhance implants’ long-term stability. However, in designing an optimal implant surface, there must be a fine balance between antimicrobial activity and the desired osteoconductive properties [31]. The balance is difficult to achieve as greater surface roughness promotes firmer bone fixation but is directly proportional to bacterial retention, which may promote biofilm formation in the long run [33].

Stainless steel is known for its good ductility and high strength [34]. However, these implants stimulate bacterial colonization when a fibrous fluid-filled capsule forms at the implant–bone interface, leading to high infection and failure rates [35]. As stainless steel implants can be difficult to integrate with soft tissue or bone, they are now mostly used in fracture fixation devices or temporary implants that will later be removed from the body [34]. Cobalt-based alloys have material properties, making them suitable for permanent implant placement. This material has higher corrosion resistance and higher hardness and strength. With these properties and the fact that cobalt is harder to configure by machine and has lower ductility, it is more commonly used in joint or hip prostheses that require high wear resistance [34,36]. Thus, although stainless-steel materials and cobalt-based alloys were used in the early 1900s [2], titanium is the predominant dental implant material used today [37], due to its superior mechanical and physical properties compared to materials used in the past [38]. Aside from titanium, zirconia and polyether ether ketone (PEEK) are also considered as dental implant materials in clinical settings today [39]. Therefore, this article aims to gather the information on surface modification development and their impact in the context of osseointegration and biofilm formation in dental applications focusing on titanium, zirconia, and PEEK materials.

## 2. Titanium Implants

With several advantageous properties, especially the excellent biocompatibility, titanium gained popularity and is now considered the metal of choice for dental implants [35]. Titanium’s biocompatibility can be attributed to the formation of the stable, self-limiting oxide layer on the surface [38], which prevents the titanium materials from further oxidizing and corroding. This corrosion-resistance characteristic facilitates biocompatibility by maintaining the mechanical integrity of the material and the health of the surrounding tissue [40]. In addition, titanium is stronger, lighter, and has a lower modulus of elasticity (110 GPa) compared to stainless steel (210 GPa) [41]. It is most favorable for a material’s Young’s modulus of elasticity to be equal to or as close to that of cortical bone (30 GPa) [42]. Besides, titanium has a large resistance to repeated loads and is less rigid, which decreases the amount of stress on the bone. Thus, the desired mechanical properties also make titanium an ideal material for use in surgical implants. Moreover, recent studies indicated that, compared to cobalt, vanadium, aluminum, chromium, and iron, titanium has more intrinsic antibacterial potency against *Porphyromonas gingivalis*, *Prevotella intermedia*, and *Actinobacillus actinomycetemcomitansi,* among others [43].

It is worth noting that several obstacles exist for titanium in contemporary dental implantology. For instance, the traditional, unmodified implant surfaces lack the full capacity to consistently achieve osseointegration and combat biofilm formation. The titanium surface of an implant is reactive when exposed to biological fluids (e.g., saliva and blood) or air, forming titanium dioxide which serves as a passivation layer that determines the biocompatibility of the implant. However, the electrochemical/physicochemical properties for titanium dioxide are fundamentally different compared with the mineralized dental hard tissues, which appear to create a distinct microenvironment that impacts the adherence oral early colonizers (such as oral *Streptococci* and *Actinomyces*) and may alter interspecies interactions associated with peri-implant health [26,27]. Furthermore, recent clinical evidence suggests that local dissolution products/particles released as a result of the implant surface degradation can trigger pro-inflammatory immune responses [28,44]. In conquering these hurdles, a diversity of physical, chemical, and biological modifications has been applied to the surface of titanium implants to enhance their biological performance and osseointegration outcomes (Table 1).

### 2.1. Physical Modifications

Physical modifications can be categorized at the macro, micro, and nano levels [54,108]. Macro-level modifications are defined by visible geometry ranging on a millimeter scale. Features such as implant body shape and differing thread patterns can improve primary fixation and long-term implant stability [109]. Micro-level modifications can increase the implants’ surface area on a micrometer scale, enhancing fibrin matrix formation as an osteoconductive scaffold for osteogenic cells and bone matrix deposition [110]. Nano-level modification can increase the implant’s surface roughness and wettability, as well as surface free energy, for enhancing cell growth and osteoblastic differentiation [111]. Ideally, an implant surface should inhibit biofilm formation but still support optimum osseointegration in vivo. Theoretically, increased surface roughness for better osteogenic potential is also associated with higher risks of biofilm formation [112] since the intensified surface roughness also leads to a more stabilized microbe-substrate linkage by increasing the surface area for bacterial attachment and protecting bacteria from fluid shear forces [65,113]. It must be noted that the susceptibility to disease progression may be higher for some moderately rough implant surface compared to others [114]. However, certain topographies, usually achieved on a nano scale, have been found to decrease bacterial adhesion and limit the establishment of infection [31]. In general, roughened surfaces have been shown in clinical studies to lead to greater implant success rates [115]. In particular, osteoblasts are sensitive to surface roughness and demonstrate a more differentiated phenotype on rougher surfaces, which results in a shortened required healing period before loading [116].

#### 2.1.1. Macro-Level Modifications

The macro-level design of dental implants takes shape (tapered or parallel), thread pattern, and overall macro-level irregularities into consideration [50]. These visible features of an implant are defined as fixture design which predominantly determines the assessable implant surface area [117,118] and the mechanical interlocking between the implant and the bone, achieve the primary stability at the implant placement, and govern the spaces for bone in-growth [119,120]. Thus, along with appropriate implant drilling preparation, the macro-level geometry of an implant is fundamental to the success of placement [121].

##### Shape

The parallel implant design, where the diameter is consistent along the implant body’s length, is one of the longest-used designs to achieve initial implant stability [122]. However, a retrospective clinical study suggests insertion of this shape implant is known to be technique sensitive [123]. In the 1990s, Dr. Jack Hahn realized that tapered implants mimicking a tooth root (in other words, those with a decrease in diameter toward the apex) could divert forces toward the apex and distribute occlusal forces to neighboring bone better than parallel implants. Thus, he developed the first tapered implant, which is more favorable for both immediate placement and immediate loading [45]. Further, with a tapered apex, less bone volume removal is needed, which is superior for an implant site with root proximity [124].

##### Diameter and Length

An objective standard for implant stability, the implant stability quotient (ISQ), whose values range from 1 to 100, with a higher ISQ value indicating greater implant stability [46], can be measured through resonance frequency analysis, a technique established by Professor Neil Meredith in 1998 [125]. Comparing 10- and 13-mm implants, constant in diameter, yielded no statistical difference in ISQ value in D1 bone, classified by Misch as dense cortical bone. Differences were only apparent when placing implants in low-quality bone or D3 bone, classified by Misch as porous cortical and fine trabecular bone. On the contrary, when comparing various implant diameters with the same length, statistically, the wider diameter of the implant, the higher the ISQ value [126]. Moreover, increasing implant diameter improves the force distribution, reduces the intensity of stresses along the entire implant length, and in turn elevates the load-bearing capacity of the prosthesis [47]. Thus, it is broadly accepted that an implant’s diameter has a greater impact on stability than length.

##### Threads

Threading on dental implants aims to achieve the most primary contact, improve primary stability, and aid with stress distribution in the bone [50]. As bone responds more favorably to compressive forces than shear forces, thread designs should minimize the shear force upon placement [120]. The angle between the face of a thread and the plane perpendicular to the long axis of an implant is called “face angle,” which is a specific thread feature governing the shear force. As the thread’s face angle decreases, the shear force decreases [119]. The thread shape and thread pitch also influence stress transfer. Among square shape, V-shape, buttress, and reverse buttress threads, V-shape threads achieve the most stability and least amount of stress. Thread pitch is defined as the distance between two neighboring threads when measurements are taken parallel to the thread axis [48]. When there is a lower pitch (in other words, more threads per unit length), each individual thread contributes less stress. Moreover, reducing pitch increases the bone-to-implant contact surface (aka. functional surface) area, further contributing to a favorable stress distribution and better primary stability achievement [127]. Meanwhile, increasing thread depth and width can also improve primary stability by enlarging the functional surface, however, this makes implant insertion more challenging [128]. In response to this challenge, double and triple thread implants have been created recently to assist implant insertion by expediting thread penetration while producing less heat during placement. This is beneficial for primary stability, and these implants ultimately are in tighter contact with the bone [50].

Meanwhile, Bermejo et al. recognized that the biofilm microcolonies were mostly deposited on the lateral surfaces and peaks of the implant threads, with little deposits on the area between threads [129], revealing that the thread structure has an impact on biofilm formation. Thus, future studies should also consider the optimal thread design to limit biofilm formation.

In summary, at the macro level, manufacturing a wide, taper-shaped, V-shape thread implant would most likely achieve more favorable clinical outcomes.

#### 2.1.2. Micro-Level Modifications

Physical modifications to implant surfaces at a micro scale often range from 1–100 μm. These micromechanical changes lead to improved secondary integration, including bone growth, turnover, remodeling, and overall interlocking of bone at the implant interface [50,120]. In particular, bone growth is encouraged at the cellular level, as microroughness attracts differentiating osteogenic cells [32]. The micro-level modified surface encourages platelets to secrete various mediators that help stabilize the blood clot and form the fibrin matrix on the implant. In turn, this fibrin matrix plays the role of an osteoconductive scaffold for osteogenic cells to migrate to, eventually leading to bone formation at the surface of the implant [119]. The surface features created at a micro scale can make a significant impact on the biofilm formation as they have a similar topographical size as the colonizing microorganisms (~1 μm), while the physicochemical properties (e.g., roughness and wettability) can also be dramatically changed. The most common types of micro-level modifications include machining, grit-blasting, and sandblasting combined with acid etching.

##### Machining

Implants with a turned, milled, or polished surface are considered the first generation of dental implants, mostly used until the 1990s [32,50]. While these machined implant surfaces appear smooth to the naked eye, slight grooves and ridges created during the manufacturing process are detectable with a scanning electron microscope (SEM) [54]. These slight surface imperfections, with an average roughness of 0.96 μm [49], effectively promote osteogenic cells to attach and deposit bone matrix [32]. Indeed, a firmer bone fixation is achieved with a surface roughness of 1–1.5 μm compared to a smoother surface [130]. Thus, several surface modification methodologies, such as grit blasting, sandblasting and acid-etching, were developed to further roughen the surface and increase the bone-to-implant contact and fixation compared to the early machined implants [131].

##### Grit-Blasting

To further roughen the surface of an implant, titanium dioxide particles of 25–50 μm are often projected toward the implant surface at a high velocity [51]. The roughened surface of grit-blasted implants results in higher success rates compared to machined implants [50]. In a ten-year follow-up clinical study [132], titanium dioxide grit-blasted titanium implants were found to have a 96.9% survival rate and were shown to have a better prognosis than those with a relatively smooth machined surface, particularly in low-density bone. Thus, this type of surface modification leads to more predictable long-term clinical outcomes in both the maxilla and mandible.

##### Sandblasting and Acid-Etching

Sandblasted-acid etched (SLA) implant surfaces are produced by sandblasting the surface with large grit particles (250–500 μm) to remodel the macrostructure of the implant, and subsequently etching the surface with an acid to create micro-irregularities [50]. Usually, strong acid solutions, such as HNO_3_/HF mixture or aqua regia (HCl/H_2_SO_4_ mixture), are used to remove the oxide layer and some underlying material, as well as the contaminants, from the titanium implant surface, resulting in pits and craters [49]. After the acid-etching step, the surface possesses homogenous irregularities, which results in increased functional surface area and improves bioadhesion [50]. This technique accelerates osteoblasts’ attachment, enhances osteoblastic retention. And ultimately facilitates successful osseointegration and more bone apposition. A minipig study showed that, in comparison with acid-etching only or machined implants, SLA implants led to more bone anchorage [52], suggesting that these types of implants should be considered specifically when a patient requires early loading of the implant [133]. Meanwhile, a long-term survival rate of 99.2% of SLA-modified implants is also reported in a clinical trial [134]. Therefore, surface topography at the micro scale is able to significantly improve osteointegration, which subsequently increases the implant survival rate.

Although higher micro-scaled roughness has been shown to favor osteointegration, it also impacts the bacterial attachment and biofilm formation [135]. A positive correlation between implant surface roughness and the affinity for bacterial attachment has been observed both in vitro and in vivo [53,136]. Indeed, compared to machined and acid-etched only, SLA modification induced the most severe colonization in vitro [53], likely due to the high roughness created. Interestingly, a “critical” roughness was observed when the Ra of a titanium surface was as low as 0.2 μm, since no further reduction of bacterial accumulation could be achieved by further polishing in a clinical study [136]. Therefore, applying a secondary surface-modifying approach that inhibits the bacterial colonization while preserving the surface roughness which is fundamental for osteointegration, may be beneficial to address the conundrum. It is also worth noting that while the underlying mechanism of microbial colonization is still not fully elucidated, some “micro-enabled” mechanisms have been proposed with regards to how these surface modifications can reduce the initial attachment (as reviewed in [137]). Thus, achieving the balance between osteointegration promotion and biofilm formation inhibition at the micro level is still a big question.

#### 2.1.3. Nano-Level Modifications

When an implant is modified at the nano level, the size of materials used for modifications ranges from 1–100 nm. Common nano-level modifications include laser ablation and nanocomposites, resulting in the absorption of proteins and adhesion of osteoblasts on the implant surface, thus enhancing osseointegration at both cellular and protein levels [32,50]. Some previous studies suggest that implants with nano-scale roughened surfaces may increase torque removal and bone-to-implant contact compared to those with micro-scale roughened surfaces [119]. These surfaces can increase the surface’s wettability for bone matrix and growth factors deposition. On the other hand, some other studies suggest that nano-scale topography alone may not be sufficient to ensure the strongest osseointegration, and additional micro-level roughness may be necessary [138]. It is important to note that nano-level modifications decrease bacterial adhesion and reduce infection [31], which coincides with the 0.2-μm surface roughness threshold, below which no further reduction in biofilm formation would be expected [136].

##### Laser Ablation

Laser ablation is a process to generate nano-scale channels around the implant collar area [54]. This process is advantageous to other surface modifying techniques mentioned above as it has the capacity to generate a complex and precise surface geometry with high resolution in a clean and rapid fashion [139]. Importantly, the laser-ablated implants induce significantly more bone-to-implant contact and larger torque removal values in comparison with machined surface implants [55]. Besides, the connective tissue fibers will run perpendicular to the laser-ablated implant surface, which is unique compared to the usual parallel orientation of fibers around an implant surface. The channels created on the implant appear to act as a biological seal by encouraging the attachment of connective tissue and bone and preventing epithelial down growth [32]. Moreover, an in vitro study suggested that laser-ablated titanium have significantly lower biofilm formation than machined and grit-blasted implants, as well as human enamel surface [56]. In particular, in a clinical study, the maximum effect of reduction in biofilm formation occurred when the surface was blasted orthogonally by the laser beam [56]. Thus, the orientation of the laser beam seems to be relevant for the biological interaction with biofilms, however, the underlying mechanisms remain unknown. Taken together, laser ablation not only results in fast osseointegration associated with enhanced connective tissue attachment, but also has the potential to inhibit biocontamination, which is desired for a dental implant.

##### Nanocomposite

Nanocomposite coatings have also been used on dental implants to manipulate materials’ mechanical properties to better match natural bone [139]. Various nanoparticles, including but not limited to titanium dioxide (TiO_2_), silver (Ag), hydroxyapatite (HA), pectins, cubic zirconia, ultra-nanocrystalline diamond, carbon nanotube, can be added to titanium using both physical and chemical techniques [139,140,141,142,143]. Typical physical methods include nanoparticle compaction, plasma spraying deposition, physical vapor deposition, and hot isostatic pressing [57]. Incorporation of the secondary nanoparticles can improve biocompatibility and osseointegration [58]. Moreover, by providing a cleanable surface, nanocomposite coatings allow the detachment of adsorbed salivary proteins and adherent bacteria under shear forces. Thus, nanocomposite coatings can also effectively reduce biofilm formation on the dental implant surface, as seen in a clinical study [59]. For instance, silver appears to be the most promising element in the nanoparticle coating for implants due to its broad-spectrum and prolonged antimicrobial activity [144]. Coating silver nanoparticles on titanium surfaces introduce statistically significant antibacterial features against *Staphylococcus aureus*, assisting in preventing peri-implantitis [145]. However, it is important for these particles to be properly fixated to the surface, as safety concerns have been raised over the usage of silver nanoparticles due to the cytotoxicity and potential hazard [146]. To address this issue, several modified approaches were proposed by using optimized nanoparticle coating techniques or making a hybrid composite that stabilized the structure [145,147], yet further investigations particularly in vivo biosafety evaluations are needed.

In summary, nano-level implant surface modifications have a promising potential in promoting osteointegration and preventing biofilm formation. A combination of modifications at different levels may provide advancing benefits, although the investigations in this area are still in infancy.

### 2.2. Chemical Modifications

Several chemical modifications have been performed on implant surfaces to improve osseointegration and mitigate biofilm formation by providing a hydrophilic surface. The most common chemical modifications used currently are led by discrete crystalline deposition, anodic oxidation, and ultraviolet (UV) treatment or photofunctionalization [54,66,69]. Overall, a chemical-modified implant surface could significantly benefit bone healing [8,148].

#### 2.2.1. Hydrophilic Implants

On a regular titanium implant, the absorption of carbonates and hydrocarbons from the surrounding air results in low surface energy, and surface roughness leads to hydrophobicity [32]. To prevent surface contact to air, minimize the absorption of carbonates and hydrocarbons, and encourage hydrophilicity, the implants have to be hydroxylated (or hydrated), rinsed under nitrogen protection, and stored in an isotonic saline solution until their use [60]. On the other hand, most chemical modifications result in a structural change at the nano-scale and create a hydrophilic surface with high surface energy [149], which elevates the amount of oxygen absorbed while reducing the amount of carbon [150]. A simple strategy is to apply a hydroxide ion solution to the implant surface to elevate the surface energy and hydrophilicity [151]. For example, when an SLA implant is rinsed under nitrogen protection to prevent exposure to air and then stored in a sealed glass tube containing isotonic NaCl solution, a SLActive^®^ (Straumann Holding AG, Basel, Switzerland) surface is achieved, resulting in a higher degree of hydrophilicity and higher surface energy [60,152]. Moreover, the wettable surface yielded from chemical modifications has a greater affinity for proteins and is capable of maintaining the proper conformation and function of the absorbed proteins, and subsequentially encouraging cell attaching and migrating onto the implant, as well as promoting osteoblast differentiation and maturation [61]. Animal studies revealed that implants with hydrophilic surfaces have a higher affinity of the initial blood clot, enhanced neoangiogenesis, better bone-to-implant contact, higher bone density, and thus benefit the earlier stages of osseointegration [62,63]. Echoing these animal studies, in human clinical studies, a stronger bone response was observed on SLActive^®^ surfaces in comparison with SLA surfaces during the early healing phase in a clinical trial [152]. It is worth noting that the later biological response was very similar between the two surfaces [152]. Thus, if SLActive^®^ implants are truly superior to SLA implants in the long term should be further assessed in detail [18].

In general, the preference of hydrophilic/hydrophobic surfaces differs among the bacterial species and can be greatly affected by other factors such as the presence of salivary pellicle and the presence of external shear stress [153]. It has been observed that bacteria with hydrophilic properties prefer to attach to hydrophilic surfaces, while those that are hydrophobic prefer hydrophobic surfaces [113]. Thus, the increased hydrophilicity from the chemical modifications discourages hydrophobic interaction and creates repulsion between the hydrophobic bacteria and the implant surface, thus preventing their adhesion and activity [64]. An in vitro study suggests that periodontal pathogens such as *P. gingivalis* exhibit hydrophobic activity [154], and therefore are less adherent to hydrophilic surfaces [155]. Similarly, *A. actinomycetemcomitans* and *F. nucleatum* also have reduced levels on hydrophilic surfaces in the culture medium [155]. Despite the complexity of bacteria-surface interaction, recent research showed that both superhydrophobic and superhydrophilic surfaces could reduce bacterial adhesion and inhibit biofilm formation [156,157], and therefore have potential applications to improve the antifouling properties of dental implants.

Thus, chemical-modified hydrophilic implants may shorten the healing period by promoting the early osteointegration while inhibiting hydrophobic bacterial adhesion. Nevertheless, the long-term benefits of hydrophilic implants are questionable, and whether this modification could effectively reduce the peri-implantitis-related biofilm formation is still unknown.

#### 2.2.2. Discrete Crystalline Deposition

Discrete crystalline deposition (DCD) is a sol-gel process where a double acid-etched surface is modified with calcium phosphate particles of 20–100 nm [66]. After this process, about 50% of the surface is composed of these calcium phosphate particles. DCD-treated titanium implants have better osteoconduction compared to commercially pure titanium (cpTi) and Ti6Al4V controls and display increased bone-to-implant contact in animal models due to an enhancement in surface nanotopography [67]. Clinically, this type of surface modification, as seen in the NanoTite™ (BIOMET 3i, Palm Beach Gardens, FL, USA) Tapered Implant, has shown encouraging results with immediate loading protocols [68]. A prospective one-year clinical study showed that NanoTite™ Tapered Implants have a one-year survival rate as high as 99.4%, with mean marginal bone resorption of 1.01 mm during the first year in function [68].

Compared to machined and acid-etched implant surfaces, DCD-modified surfaces showed a significant reduction in bacterial attachment [65]. Specifically, through total viable count and confocal laser scanning microscopic methods, adhesion of *A. actinomycetemcomitan*, *S. mutans*, and *S. sanguis* was significantly reduced on DCD-modified surfaces in comparison with acid-etched surfaces [65], which can be attributed to the decrease in surface roughness of this material, as measured by a laser profilometer and SEM.

#### 2.2.3. Anodic Oxidation/Anodization

Anodic oxidation is a process that modifies the titanium implant surface electrochemically to create a thicker TiO_2_ layer [69]. Usually, the TiO_2_ layer is 17–200 nm thick on the surface of an unmodified titanium implant, while the anodic oxidation process can expand the TiO_2_ layer to 600–1000 nm [69]. Besides, this anodic oxidation-induced thicker TiO_2_ layer contains various porosities, encouraging gingival fibroblast deposition, adhesion, and proliferation, as well as osteoblast adhesion [32,50]. Thus, the modified surface is osteoconductive [32]. For example, at 16 weeks after implantation in monkey type IV bone, classified by Lekholm and Zarb as very thin cortical bone with low-density trabecular bone of poor strength, the anodized titanium implant displayed a 74% bone-to-implant contact [70]. A clinical study showed anodized implants had a 100% survival rate without any sign of infection or pathologic process, compared to 96.4% for turned titanium implants [158]. Another study suggested a 10% higher success rate of anodized implants than machined implants following immediate loading [71]. Furthermore, anodization of titanium significantly reduced the number of adhered bacteria by 1–2 logs [159], which could be sufficient to prevent the establishment of infection in an environment where the bacterial inocula are low, such as in surgical procedures [31], but perhaps would not overcome the challenge of high bacterial inocula or in a patient with a compromised immune system.

#### 2.2.4. Fluoride Treatment

When implant surfaces are modified with fluoride ions, bone formation is enhanced as fluoride stimulates undifferentiated osteoblasts and osteoprogenitor cells to proliferate, differentiate, and have increased alkaline phosphatase activity [54,72,73]. Moreover, fluoride encourages bone mineralization by attracting calcium deposition [160]. Placing the titanium implant in a hydrofluoric acid solution of an electrochemical cell is the process used to make the fluoride deposit on the implant surface. In the redox system, the fluoride implant acts as the cathode, while the fluoride ion gives an electron to the titanium ion causing reduction of the titanium [74]. Thus, the process of fluoride treatment is indeed a cathodic reduction reaction. Since a high concentration (1 mM) of fluoride ions is cytotoxic [72], only a very low density of fluoride can be deposited on the implant surface [161], while such a low fluoride density is sufficient to promote bone binding to the treated implants in comparison with those without fluoride deposition. For example, comparing with grit-blasted implants, fluoride-treated implants showed more firm bone-to-implant contact and greater removal torque [75]. Meanwhile, fluoride ions in saliva can act as a lubricant due to the precipitation of calcium fluoride salts. For over a century, fluoride has been used to improve oral health for its activity that protects dental hard tissue and antimicrobial properties while the impact of incorporating fluoride on titanium implants is still not elucidated. Nevertheless, fluoride was shown, in a clinical study, to impair the corrosion resistance of titanium, which should be considered when applying such modifications [162]. Thus, the presence of fluoride on titanium implant surface may facilitate the structural disruption and detachment of biofilm [76], while further investigations focusing on the dental implant microenvironment are warranted.

#### 2.2.5. Hydroxyapatite (HA)

HA can serve as a source of both calcium and phosphate for bone formation [32]. Since HA is a major component of bone tissue, when a titanium implant is coated with HA it gains an osteoconductive character [54]. To date, Nano-HA is one of the most used materials for titanium coating [163]. The most common way to achieve this modification is through plasma spraying [164], in which a plasma flame that contains HA particles is heated to approximately 15,000 Kelvin and sprayed onto the titanium implant surface [165]. The physical and chemical properties of the coated HA layer can be modified by different spray parameters, such as gas combination and spray flow rate, power, and stand-off distance [166], while the optimal thickness of the HA layer is 40–50 μm [77]. Previous clinical studies showed that HA-coated implants could be beneficial in an environment where more rapid bone-to-implant contact is needed, such as in grafted bone or type IV bone (very thin cortical bone with low-density trabecular bone of poor strength) [167]. Interestingly, electrochemical deposition of fluoridated hydroxyapatite may also have antibacterial effects against *S. aureus* and *P. gingivalis* specifically [78]. However, the long-term stability and clinical outcomes of HA-coated implants are still uncertain. Rams et al. showed in a clinical study that HA-coated and uncoated titanium implants did not display significant clinical differences regarding osteointegration and microbial contamination [87]. Besides, Albertini et al. indicated in a clinical study that implants with failure of HA-coating adhesion exhibited more bacterial microleakage and peri-implant tissue complications [168,169,170]. Moreover, coating failure attributed to the thickness of the HA layer can lead to larger peri-implant defects [171]. Hence, the current understanding of HA coating seems to be controversial, which should be fully evaluated in the future.

#### 2.2.6. UV Treatment/Photofunctionalization

UV treatment (aka, photofunctionalization) enhances osteoconductivity of the titanium implant surface by altering the hydrophilicity of the TiO_2_ layer [172]. Specifically, UVA (320–400 nm) and UVC (200–280 nm) waves can increase the hydrophilicity of the titanium surface and thus enhance osteogenic cell attachment and proliferation and plasma protein adsorption [54]. It has been found that this modification mostly promotes bone formation in the early phase of osseointegration [79]. When comparing UVA-treated titanium implants to untreated ones in a dog model, bone-to-implant contact was significantly enhanced two weeks after healing, but not after four weeks [79]. Similarly, in human studies, UV treatment enhanced the early phases of osseointegration of compromised bone, as well as in the situation that required an early loading protocol [173,174]. Moreover, photofunctionalization has been shown to have antibacterial effects of prohibiting biofilm formation [148]. Del Curto et al. showed that anodized-heat-treated titanium followed by UV light treatment exhibited a reduction in the attachment of *S. mutans*, *S. salivarius*, and *S. sanguis*, while enhancing cellular metabolic activity, including bone cell proliferation and differentiation [80].

#### 2.2.7. Plasma

Plasma in physical science is defined as the fourth state of matter, while in biology, it refers to the non-cellular fluid portion of blood [175]. Irving Langmuir coined the term plasma in 1927 when he noted that ionic liquids recognized as plasma in biology have characteristics similar to the plasma spoken about in physical science, concerning the way blood plasma carries red and white blood cells and electric fluid carries electrons and ions [176]. The use of plasma in medicine is constantly evolving, and various techniques have been applied to dental implantology to functionalize titanium’s surface and improve its biocompatibility [81].

##### Atmospheric Pressure Plasma Processing

Atmospheric pressure plasma processing can be used to increase the hydrophilicity of the material and therefore encourage cell adhesion to the surface [177]. Using a piezobrush to achieve atmospheric pressure plasma treatment on a pure titanium surface increased cell adhesion, alkaline phosphatase activity, and factors related to bone differentiation [81], suggesting effectively enhancing osseointegration without altering the other beneficial titanium surface properties. Similarly, treating titanium surfaces with non-thermal atmospheric pressure plasma jet (NTAPPJ) significantly increased the hydrophilicity and surface energy of titanium without changing the surface’s topographical features [82]. Moreover, bacterial adhesion and biofilm formation rate were significantly reduced on these treated surfaces. Particularly, the reduction was more significant for Gram-negative bacteria, potentially due to their specific cell wall structure being more sensitive to oxidation caused by NTAPPJ [82].

##### Plasma Oxidation

The plasma oxidation methodology also can be used to increase the wettability of the titanium surface. For example, using a radio frequency plasma-enhanced chemical vapor deposition system to archive plasma oxidation on SLA titanium implant, a super-hydrophilic surface was created on the plasma-oxidized SLA (PO-SLA) surface [83]. Consequently, the PO-SLA surface exhibited a higher removal torque and a higher bone-to-implant contact.

Thus, plasma modifications seem to have promising outcomes based on current knowledge. As a novel area of implant surface modification, whether this could be widely used clinically still needs further investigation due to the lack of in vivo analysis.

#### 2.2.8. Calcium Chloride Treatment

When titanium undergoes a hydrothermal treatment with calcium chloride (CaCl_2_), osseointegration and the soft tissue seal were improved on the yielded CaCl_2_-treated titanium (Ca-HT) surface [19]. Meanwhile, the adsorption of laminin-332 and osteopontin and adhesion of osteoblasts on the Ca-HT surface are enhanced [19]. Moreover, greater attachment of gingival epithelial-like cells and fibroblasts was found on titanium disks with this treatment [19]. On the other hand, Ca-HT treatment was proven not to affect bacterial adhesion, specifically of *S. gordonii*, suggesting that this modification is capable of promoting cell adhesion without increasing bacterial adhesion [19]. It is speculated that the calcium present on the titanium surface can influence the composition of the acquired pellicle from saliva, allowing for the enhancement of the biocompatibility of titanium without the enhancement of bacterial attachment.

### 2.3. Biological Modifications

Various biological modifications have been applied to implant surfaces as well, including plasma (in the biological sense), extracellular matrix (ECM), peptides, growth factors, messenger molecules, drugs, and antibacterial agents. These modifications have shown a diversity of effects on osseointegration and biofilm formation.

#### 2.3.1. Platelet Rich Plasma (PRP) and Platelet Rich Fibrin (PRF)

As the reservoirs of growth factors promote osteoblast adhesion, PRP and PRF can improve bone healing [84]. For instance, a clinical investigation demonstrates that applying PRP with autogenous bone or organic bone substitutes in the implant site just before the implant insertion leads to satisfied aesthetical and functional results [178]. Concerning the implant surface modification, an in vitro study showed an increased number and length of filopodia in adherent osteoblasts on the titanium surfaces treated with PRP or PRF with zoledronic acid (a bisphosphonate agent often used clinically for osteoporosis, high blood calcium levels, and the effects of cancers that have spread to the bone) compared to the surfaces treated with zoledronic acid alone [84], suggesting PRP and PRF have the potential to enhance initial bone apposition and primary stability of dental implants, particularly in patients undergoing bisphosphonate treatment. It is worth noting that the comparison between PRP and PRF on osteogenic cells is controversial [179] and limited to the in vitro cell culture level. Thus, more comprehensive studies are demanded to investigate the growth factor components, growth factor concentration, and in vivo effects of PRP and PRF.

#### 2.3.2. Extracellular Matrix (ECM)

During osseointegration, fibroblasts secrete ECM molecules, such as collagen, chondroitin sulfate, vitronectin, and fibronectin, which guide osteoprogenitor cells to the functional surface [180]. Thus, by applying ECM components to the implant surface, the cell-ECM interaction can activate various signaling pathways to enhance bone healing [85]. In particular, implants coated with collagen/chondroitin sulfate or collagen/sulfated hyaluronan have been shown to increase bone formation and maturation compared to uncoated implants [86], confirming the positive effects of ECM-coating on osseointegration.

#### 2.3.3. Peptides

##### Arginylglycylaspartic Acid (RGD)

As a binding site for integrin receptors, RGD peptide is a specific amino acid sequence that plays an essential role in osteogenic cell adhesion and migration [32]. Histomorphometrically, RGD-coated implants are comparable to those coated with larger ECM components concerning peri-implant bone regeneration measured by bone-to-implant contact and bone volume density [181]. Interestingly, Schliephake et al. showed that RGD-coating led to improved bone-to-implant contact on a polished titanium surface in beagle dogs three months post-implantation [88], while Broggini et al. did not detect any effects of RGD-coating on SLA implants regarding bone-to-implant contact, new bone fill, or removal torque values in miniature pigs two weeks post-implantation [89]. This inconstancy may suggest a particular temporal effect of RGD-coating on osseointegration.

##### P15 Peptide

P15 is a synthetic 15 amino acid peptide that imitates the cell-binding domain of the alpha-1 chain of human collagen [182]. Attaching this peptide to the titanium surface has been shown to promote osseointegration, particularly by increasing osteoblast and mesenchymal cell attachment, spreading, and osteogenic gene expression and differentiation [90]. Interestingly, when P15 was fused with competence-stimulating peptide (CSP), the fusion peptide displayed the osteogenic activity and suppressed biofilm formation [183]. These novel potencies are not shared by P15 and CSP alone, although CSP is a cationic antimicrobial peptide inhibiting *S. mutans* planktonic growth [184].

##### Strontium-Incorporated Protein

Coating with strontium-incorporated protein significantly improved bone-to-implant contact, bone formation, and biomechanical properties [91]. In particular, a strontium-containing phase change lysozyme coating enhanced early adhesion, proliferation, and osteogenic differentiation of bone marrow stromal cells, increased the expression of osteogenic related genes, such as *bone morphogenetic protein 2 (BMP-2)*, and had a significant capability of new bone formation in vivo [92]. While various techniques can coat strontium-incorporated protein onto the titanium surface, such as the magnetron sputtering process and hydrothermal treatment, the coating method does not appear to affect bone implant contact percentages [93].

##### Bactericidal Peptides

Various bactericidal peptides, such as GL13K and human beta defensins (HBDs), have been used to exert an antibacterial effect on implant surfaces. GL13K is derived from a parotid secretory protein, BPI fold-containing family A member 2 (BPIFA2), a defense protein found in saliva [185]. Holmberg et al. showed that applying GL13K to implant surfaces has a bactericidal effect against *P. gingivalis*, while maintaining cytocompatibility with the adequate proliferation of osteoblasts and gingival fibroblasts [94]. Similarly, when HBDs, a group of peptides that exert antibacterial effects on epithelial borders, were applied to implant surfaces, they exhibited broad-spectrum antibacterial functions and maintained the proliferation of osteoblasts and mesenchymal stem cells [95].

##### Sclerostin-Antibody

Sclerostin is an inhibitor of the Wnt/β-catenin signaling pathway that regulates bone growth [186]. It has become a therapeutic target for treating osteoporosis and is currently under study in dental implantology for its role in osseointegration [187]. Interestingly, expression of sclerostin and RANKL expression increased after an implant endures impact, suggesting sclerostin may be involved in the processes of peri-implant bone damage [188]. In addition, systemic administration of a sclerostin-neutralizing antibody was found to repair bone defects surrounding dental implants in an experimental alveolar bone osteotomy rat model [189]. The sclerostin antibody has also been studied as conjugated to a TiO_2_ nanotube array, in which the decrease of sclerostin was found to promote osteoblast differentiation [96]. Although this study suggests that lower levels of sclerostin could improve a dental implant’s biomedical performance, particularly in osteoporotic conditions, specific clinical studies using sclerostin-antibody for titanium surface modification are lacking.

#### 2.3.4. Growth Factors

Platelets and macrophages, present in the first phase of osseointegration, release various growth factors that help initiate the second phase of osseointegration [180]. These growth factors, including transforming growth factor β (TGFβ), platelet-derived growth factor (PDGF), and fibroblast growth factor (FGF), all have been applied to implant surfaces to jumpstart this process.

##### Bone Morphogenic Proteins (BMPs)

Within the TGFβ family, specific BMPs, such as BMP-2, BMP-4, and BMP-7 play a role in stimulating bone formation [190]. It has been clearly shown that BMP coating on titanium implant surfaces promoted bone regeneration [88]. In particular, BMP-2-containing titanium implants displayed an increased density of surrounding bone compared to acid-etched ones, and have better bone-to-implant contact and new bone formation compared to anodized implants [98,99].

##### Platelet-Derived Growth Factor (PDGF)

When PDGF is released, it enhances new bone deposition by encouraging collagen synthesis and enhancing bone matrix deposition [191]. In osteoporotic rats, implants coated with PDGF enhanced osseointegration [100]. Moreover, PDGF-coated titanium implants accelerated soft tissue healing around the implant surface [101].

##### Fibroblast Growth Factor (FGF)

Fibroblast growth factor, specifically FGF-2, directly influences the proliferation of osteoblasts [192]. When FGF-2 nanoparticles were coated on titanium materials through electrospray deposition, cell spreading and differentiation in vitro were increased [102], and the titanium implant’s osseointegration in rabbit tibia in vivo was boosted [102].

#### 2.3.5. Drugs

##### Statins

Drugs of the statin family, such as simvastatin, are most commonly used to lower cholesterol; however, their application in implantology has been discussed recently [50]. In particular, simvastatin may enhance the bone-to-implant contact and promote bone formation by stimulating the expression of the BMP and vascular endothelial growth factor (VEGF) [193]. Simvastatin-loaded porous titanium surfaces increased alkaline phosphatase activity, type I collagen synthesis, and osteocalcin release from pre-osteoblasts in vitro [103], suggesting that simvastatin-loading accelerated osteogenic differentiation of pre-osteoblasts. However, it must be noted that more standardized studies with less risk of bias are needed to more precisely clarify the role statins may play in osseointegration [193].

##### Bisphosphonates

Coating bisphosphonates, a group of medicines commonly used for osteoporosis treatment, on dental implants may positively influence osseointegration, as measured by removal torque, bone-to-implant contact, and new bone formation [194]. In a randomized clinical trial, dental titanium implants coated with a fibrinogen layer containing two bisphosphonates, pamidronate disodium, and ibandronate, significantly strengthened the mechanical fixation compared to untreated titanium [104]. However, controlled release of bisphosphates from the implant surface may be difficult to achieve, and to date, there are no commercially available systems that provide site-specific, sustained bisphosphonate release [50,195]. Moreover, bisphosphonates-coating on implant surfaces may potentially escalate bacterial adhesion and biofilm formation, as previously observed in bisphosphate-coated HA discs [105]. As the bacterial attraction effect may be attributed to bisphosphonates’ chemical structure that displays a direct electrostatic interaction with bacteria [105], this disadvantage of bisphosphate-coating may also extend to bisphosphonate-modified titanium implants.

##### Antimicrobials

There are two distinct types of antimicrobial surfaces that have been created in dental implantology to date. Type I surface actively elutes antimicrobials to prevent bacterial adhesion and promote killing, while type II surface consists of permanently bonded agents that can avert long-term bacterial adhesion [31]. Implants with type I surface have been successful in treating implant-associated infections; however, their eluting activities could be problematic as the initial burst of antibiotic is achieved in the first week of placement and exponentially declines over time, associated with the risk of raising antibiotic-resistant bacteria that will survive for a long period [196,197]. To overcome this drawback of the type I surface, various bactericidal and bacteriostatic agents were permanently applied to implant surfaces for building a type II surface for reducing biofilm formation around dental implants [198]. For example, permanently coating tetracycline, a bacteriostatic agent, to implant surfaces effectively killed microorganisms that would otherwise contaminate the implant surface and thus promoted cell proliferation and bone healing [106]. Moreover, when tetracycline was permanently applied to machined, sandblasted, and anodized surfaces, the surface microstructures that benefit cell integration did not change [199]. Similarly, when titanium implants were coated with vancomycin in a permanent fashion, colonization by *S. aureus* was inhibited while bone-healing was boosted [107]. Vancomycin, which reversibly interacts with bacteria, is able to be repeatedly challenged on a titanium surface. Thus, as an agent for implant coating, vancomycin is superior to the antibiotics that irreversibly bind to bacteria, such as gentamicin, whose activity declines when re-challenged [198]. However, the broad application of vancomycin will worsen the concern of the raising of vancomycin-intermediate *S. aureus* (VISA) and vancomycin-resistant *S. aureus* (VRSA) strains [200].

In summary, a variety of biological modifications have been proposed to potentially increase the implant survival rate, especially in compromised sites. However, how to control the release of the medications or proteins to achieve maximum medication activity while maintaining the physical properties of the implants and minimizing side effects remains a technical challenge.

### 2.4. Surface Cleaning of Titanium Implants

In the scenario of peri-implantitis, complete re-osteointegration cannot be achieved without surface decontamination. Thus, various surface cleaning methods have been employed, including mechanical debridement, chemical decontamination, and photodynamic therapy [201,202]. It is worth noting that implant surface modification could affect the selection of surface cleaning methods. For example, among the mechanical debridement, ultrasonic scalers with a metal tip work more thoroughly on a polished surface, while ultrasonic scalers with a non-metal tip lead to a better outcome on smooth and SLA surfaces. Metal curettes and rotating titanium brushes could be applied on SLA surfaces. The air abrasive was effective in cleaning machined, SLA, and plasma-sprayed surfaces [203], while current research shows HA-blasting/acid-etching surface response in a more excellent manner to the air-flow erythritol method than SLA surface [204]. In addition, the implants with high thread pitch and apically facing threads represent low decontamination effectiveness in the surface cleaning with titanium brush and metallic ultrasonic tip [205]. While, for the chemical decontaminations, citric acid is not proper for cleaning smooth or plasma-sprayed titanium implants, but it works efficiently on machined and HA-coated implants [202]; while chlorhexidine might work adequately on plasma-sprayed or SLA surfaces [201].

On the other hand, since several surface cleaning methods are also used for surface modification as described above, these cleaning methods could alter the implant surface. For example, airpower abrasive treatments could cause small crater-like defects, rounding, or removal of sharp edges [201,203,205]. Er:YAG laser microexplosions can remove layers of titanium dioxide from contaminated rough implant surfaces, while due to the thermal effect, Er:YAG laser could also induce melting down of peaks for SLA titanium discs and form cracks on polished titanium surfaces [202].

Overall, since some implant surface medication strategies are still in their infancy, there is no doubt that more comprehensive research on the effects of surfacing cleaning methods on different modified implant surfaces is warranted.

## 3. Materials Other Than Titanium

While titanium has several properties that deem it superior to previous materials used in implantology, its use has some disadvantages. First of all, because titanium’s elastic modulus is greater than bone, patients have experienced bone resorption and implant fracture [206]. Further, titanium is associated with scattered radiation, surface degradation, and, occasionally, allergies, all of which are harmful to the surrounding tissues [207]. In particular, a type IV allergic reaction directed toward the implant can arise when cyclic loading and the acidic oral environment lead to surface oxide layer breakdown and metallic ions release [208]. Titanium, with its dark gray coloration, can also present with an esthetic concern, especially in anterior regions of the mouth, with thin gingiva, gingival recession, or if a translucent ceramic restorative crown is used and metallic show through occurs [209]. Thus, several other materials, such as zirconia and PEEK, are currently being investigated to continuously improve patient implant outcomes [6,8,206].

### 3.1. Zirconia

Due to esthetic concerns that may arise with soft-tissue recession and the use of dark gray titanium implants, zirconia was introduced to dental implantology 2005 [210]. As a tooth-colored material, zirconia can improve the patient’s esthetic outcome, both at the soft tissue and restoration level [211,212]. Meanwhile, similar to titanium, zirconia is osteoconductive and biocompatible, while it causes less tissue reaction and toxic ion releasing to the surrounding tissues [38,119,213]. In particular, the alloy of zirconium with yttrium, which forms a stable structure of yttrium tetragonal zirconia polycrystal (Y-TZP) at room temperature, displayed advantageous biological and mechanical properties [119,213] to stimulate osteogenic cell proliferation during osseointegration as it has high fatigue resistance, elastic modulus, fracture toughness, bending strength, corrosion resistance, and low-temperature degradation [206,213,214]. Both animal and human studies demonstrate the deposition of newly formed mature bone in close proximity to zirconia implant surface with few marrow spaces, minimal inflammation, and numerous small actively secreting multinucleated osteoblasts [215]. Moreover, zirconia implants display a comparable bone-to-implant contact value with titanium implants [215]. Thus, zirconia implants have similar osteointegration with their titanium counterparts, at least in the early healing stage [216]. Moreover, zirconia implants are associated with less biofilm formation than titanium implants, decreasing the risk of peri-implantitis [217]. However, the disadvantage of low-temperature degradation of Zirconia needs to be noted when choosing the implant design [218]. As zirconia is an inert biomaterial, several techniques have been applied to its surface to improve its mechanical behavior and biological response [213] (Table 2).

#### 3.1.1. Physical Modification of Zirconia

##### Machined

Machined zirconia implants have been found to have comparable performance to machined titanium implants as the machined zirconia having a bone-to-implant contact value of 33.74–84.17%, while the value of machined titanium is 31.8–87.95% [213]. However, it is interesting to note that the cell proliferation of osteoblasts on machined titanium surfaces is significantly greater than that on machined zirconia [219], while machined zirconia-based surfaces had a statistically significant decrease in biofilm thickness compared to machined titanium [217].

##### Acid-Etching/Sandblasting

Similar to titanium, zirconia surface can be grit-blasted and acid-etched to increase the surface area for osseointegration. Particularly, Qahtani et al. showed that zirconia sandblasted with 250 μm sized particles resulted in the most enhanced osteoblast cell adhesion in vitro [219]. Besides, grit-blasted zirconia surfaces with or without acid-etching significantly prohibited *S. sanguis* and *P. gingivalis* biofilm formation [220], which is contrary to the previous observation on the SLA titanium surface [229] and highlights the advantage of zirconia materials.

##### Laser Modification

Laser modification has also been used in order to enhance zirconia implants’ osseointegration by increasing the surface free energy and wettability [230]. An in vivo study that used American Fox Hound dogs concluded that laser-modified zirconia implants could produce comparable osseointegration as SLA titanium implants concerning the bone-to-implant contact and crestal resorption at one and three months [221]. Another in vivo study in rat tibiae noted that laser-modification could increase the surface roughness of zirconia implants and thus led to significantly greater bone-to-implant contact and removal torque than machined ones [222]. Specifically, cell proliferation, phosphatase activity, osteocalcin expression, and calcification were significantly elevated on the laser-modified zirconia surfaces.

##### Coatings

Various coating materials such as silica, magnesium, nitrogen, carbon, and HA have been described to improve zirconia’s biological properties [213]. For instance, silica has been found to not only reduce bacterial adhesion, but also enhance HA formation and osteoblast proliferation on the zirconia implant surface [223]. Magnesium coatings have been shown to increase the bioactivity of implants by inducing more cell proliferation and differentiation as compared to pure zirconia-calcium phosphate coatings [224]. The addition of nitrogen and carbon has shown similar mechanical and biologic enhancement on the zirconia implants. Specifically, zirconia coated with a C:H:N layer exhibited remarkedly antibacterial properties, as they harbored less initially adherent microorganisms and undermined their vitality [225]. Similarly, HA-coated zirconia leads to a statistically significant greater volume of new bone formation and therefore enhanced osteogenesis [226].

#### 3.1.2. Chemical Modification of Zirconia

##### UV Treatment

As on titanium implants, UV-treatment on zirconia implants leads to electron excitation that increases the surface free energy, wettability, and hydrophilicity of the surface. Studies show that this treatment leads to enhanced attachment, proliferation, and differentiation of osteoblasts, as well as faster osseointegration, greater bone volume, and bone-to-implant contact [213,227,228].

Thus, with its advantages on esthetics, osteointegration, and antibacterial properties, zirconia could be a promising titanium replacement as the basic material for surface modification to gain desired dental implant outcomes.

#### 3.1.3. Surface Cleaning of Zirconia Implants

Assessments regarding the efficiency and influence of surface cleaning on zirconia implants are limited, and the majority of the available research is limited to the zirconia implant abutment instead of the zirconia implant fixture. However, it is still worth noting that, compared to titanium, zirconia is less susceptible to surface changes after curette treatment, carbon fiber reinforced plastic curette treatment, ultrasonic scaling, or air polishing with glycine powder [231,232]. Thus, there is no significant alteration concerning bacterial adhesion and epithelial attachment on the zirconia surface [231,232]. Further investigations are needed regarding the effects of surface cleanings on zirconia implants and surface-modified zirconia implants.

### 3.2. Polyether Ether Ketone (PEEK)

PEEK is a semi-crystalline linear polycyclic thermoplastic, organic synthetic polymeric material first developed in 1978 [206,208]. It has been used as a substitute for metallic components in orthopedics and trauma, as well as in dental implantology as the implant body along with the implant abutment [206,233]. It has a low density and elastic modulus (3–4 GPa), high mechanical and chemical resistance, and high-temperature stability exceeding 300 °C [234]. As its mechanical properties are more compatible with bone, PEEK may exhibit lower stress shielding compared to titanium [235]. Moreover, with its beige color and radiolucent appearance, PEEK has a more esthetic appearance than titanium and leads to fewer artifacts on imaging [235,236]. Additionally, fewer allergic reactions were caused by PEEK in comparison with titanium [237]. Moreover, PEEK is a versatile material whose bulk and surface properties can be altered to best fit its application’s requirements. While PEEK has several advantages compared to titanium, PEEK alone is bioinert, as it has low surface free energy and does not have ingrained osteoconductive properties [208]. Compared to titanium and zirconia, the PEEK implant led to low bone-to-implant contact [238]. By modifying the PEEK surface, cell adhesion, proliferation, biocompatibility, and osteogenic properties can be enhanced. Moreover, as the PEEK material can be vulnerable to biofilm formation, different modifications to the surface must be made to reduce the risk of peri-implantitis and make it more suitable for dental implants (Table 3).

#### 3.2.1. Physical Modification of PEEK

##### Roughening

Similar to the modifications discussed with titanium and zirconia, PEEK’s surface can also be acid-etched or sandblasted in order to increase the surface roughness of the material and improve osseointegration [239,240].

##### Reinforcing

A closer modulus of elasticity helps optimize the biomechanical load distribution between the implant and surrounding bone and maintain greater bone-to-implant contact [42]. As stated previously, the elastic modulus of titanium is 110 GPa [253], which is significantly higher than that of cortical bone, around 30 GPa [42]. On the other hand, the elastic modulus of PEEK can be modified to more closely match cortical bone’s elasticity by reinforcing it with different materials and changing the fiber lengths and orientations of those materials [206]. For instance, when PEEK is reinforced with carbon or glass, the elastic modulus of the resulted carbon-fiber-reinforced PEEK (CFR-PEEK) and glass-fiber-reinforced PEEK (GFR-PEEK) is increased from 3–4 GPa to 18 GPa and 12 GPa, respectively [235,254]. In addition, CFR-PEEK implants exhibited more desirable osseointegration than titanium implants regarding the bone-to-implant contact [241]. On the other hand, CFR-PEEK implants were associated with higher stress concentrations, particularly at the cervical region, while the titanium implants exhibited a more homogenous distribution [238]. Moreover, CFR-PEEK may be esthetically unfavorable due to the black color that accompanies the carbon fibers. Thus, CFR-PEEK may not be a better choice to replace titanium for dental implants.

##### Creation of Nanocomposites

Various nanoparticles can also be coated onto the PEEK surface to create bioactive nanocomposites with superior bioactivity and tensile properties compared to PEEK alone [206]. Often, HA or titanium nanoparticles are used in combination with PEEK through the process of melt-blending to enhance its mechanical properties and increase osseointegration [242]. When this technique is used in conjunction with carbon fiber reinforcement, a carbon fiber reinforced polyether ether ketone—nano-scale HA biocomposite can be formed (PEEK/CF/n-HA), the yielded surface promoted osteogenesis and encouraged cell growth around the implant surface [208]. With an increase in roughness, hydrophilicity, and Ca^2+^ ions from the HA, cell attachment and proliferation are enhanced [243]. Moreover, nano-scale fluorohydroxyapatite (HAF) can be used in conjunction with PEEK to produce PEEK/nano-HAF, leading to a greater increase in bone cell proliferation compared to HA and also possesses antibacterial properties, particularly against *S. mutans*, due to the fluoride ions in the material [242]. Therefore, this material can be beneficial for combating peri-implantitis. It must be noted that it is most beneficial to combine PEEK with nano-scale particles rather than micro-scale particles, which create inferior mechanical properties compared to pure PEEK or CFR-PEEK [242].

#### 3.2.2. Chemical Modifications of PEEK

##### Nitration, Sulfonation, Amination

Nitration, sulfonation, and amination were also used to modify the surface of the PEEK material for biocompatibility enhancement to achieve early osseointegration [206]. Moreover, sulfonation treatment can be used to incorporate lactam-based antibiofilm to establish a surface that is more resistant to bacterial contamination [208], and thus the resultant PEEK implants are highly resistant against *S. aureus* and *Escherichia coli* specifically [208].

##### UV Treatment

As on the titanium and zirconia surfaces, UV irradiation was used to make PEEK surfaces more hydrophilic with the enhanced wettability [206,242]. Additionally, UV-treated PEEK surfaces are favorable for early attachment of soft tissue and cell proliferation [244], particularly the attachment of fibroblasts for a fast and dense peri-implant soft tissue seal.

##### Plasma

Various plasma treatments can be performed on PEEK to improve its surface properties. Gas plasma, in which the implant was exposed to low power plasma gases such as water vapor, ammonia, oxygen/argon, and nitrogen, has successfully introduced several functional groups on the PEEK surface to make the surface more hydrophilic and rougher, and thus accelerate mesenchymal cell proliferation for bone formation [242,245]. Thus, in comparison with untreated PEEK implants, nitrogen-plasma-treated PEEK implants displayed greater torque removal values and bone formation, which are nearly comparable to titanium implants [246]. Meanwhile, gas plasma treatment also eliminates the chance of delamination of a surface coating from PEEK materials [255], which is an additional advantage for this dental implant surface modification.

Further, the process of plasma immersion ion implantation was used on PEEK surfaces. This technique involves putting the implant in a plasma of particles that is repeatedly pulsed with high negative voltages which accelerates and deposits ions onto the surface, creating a thin film of various particles [242]. It was found that fluorinated PEEK created through this technique not only improved cell adhesion, cell spreading, proliferation, and alkaline phosphatase activity when compared to pure PEEK, but also had bacteriostatic properties against *P. gingivalis*, a periodontal pathogen [247].

##### Coating Techniques

The methods of spin coating and electron beam deposition were used to coat the PEEK surface and improve its material properties [242]. The spin-coating process with nanohydroxyapatite involves creating a thin layer of nanohydroxyapatite by spinning the implant at high speeds and then heat-treating the PEEK surface. A thin coating layer is created through this process, which led to a higher removal torque value of the coated PEEK implants [248]. However, no significant differences regarding bone-to-implant contact were observed between the coated and uncoated PEEK surfaces [242]. Besides, electron beam deposition has been used to deposit a thin titanium coating on the PEEK surface, which advanced the surface wettability and cellular adhesion [249]. In vivo animal studies demonstrated that titanium-coated PEEK implants had significantly greater bone-to-implant contact than uncoated PEEK implants [250].

##### Antibacterial Modification

An inorganic antibacterial agent commonly used to modify PEEK implant surfaces is silver, which has a broad spectrum of antimicrobial activity against both Gram-negative and Gram-positive bacteria [240]. Long-term release of silver ions has strong bactericidal potency [251]. Previous investigations showed that PEEK materials coated with silver nanoparticles increased the success rate of dental implantation and reduced peri-implantitis [252]. Besides, titanium oxides also exhibit antibacterial properties [252]. Specifically, titanium pentoxide (Ti_3_O_5_) combined with 12.5% silver applied on PEEK surfaces has shown to be effective against *S. aureus* [251], accompanied by good biocompatibility [251]. Thus, titanium oxide could be considered an alternative for PEEK coating, with or without silver nanoparticles.

In summary, although PEEK itself may not be promising for dental implants to date, it could be largely improved to reach a comparable or even better level than titanium through the boosting development of the surface modification technologies.

#### 3.2.3. Surface Cleaning of PEEK Implants

There is no research that directly evaluates the effects of implant surface cleaning methods on PEEK. While evaluating PEEK as the candidate restorative material, among different professional cleaning methods, Al_2_O_3_ powders and airflow comfort could increase the surface roughness of PEEK [256]. Further evaluations about currently available surface cleaning strategies on surface-modified PEEK implants are needed. In addition, a special surface cleaning method, other than those applied on metal materials, might be needed for PEEK implants.

## 4. Conclusions and Outlook

The intricacies between various surface modifications differ in their balance between enhancing osseointegration through the recruitment, adhesion, and proliferation of osteogenic and fibroblastic cells and minimizing bacterial adhesion and biofilm formation. Surface roughness, which directly affects both osseointegration and biofilm formation, is the primary target for all kinds of surface modifications. However, the sweet spot is difficult to determine because a surface roughness of at least 1–1.5 μm is needed to achieve firmer bone fixation, while the bacterial retention threshold is 0.2 μm, above which an increase in bacterial accumulation occurs. Among physical modifications of titanium, micro-level modifications seem to achieve the most robust osseointegration, while nano-level modifications decrease bacterial adhesion more effectively. Regarding chemical modifications, nano-level modifications are achieved to increase the surface’s hydrophilicity and thus promote osseointegration while reducing hydrophobic bacterial adhesion. Moreover, more robust and direct stimulation of osseointegration and mitigation of biofilm formation can be accomplished by specific biological modifications. For example, growth factor-coating is known to enhance osseointegration, while antibacterial agent-coating directly combats bacteria and enhances implant properties.

Although alternative materials such as zirconia and PEEK have been studied to date, it is difficult to draw definitive conclusions about their advantages over titanium. A significant drawback of certain studies is that, with many different variables and limited direct comparisons, most comparison studies were conducted in vitro using discs, rather than in vivo, with the shape of a dental implant that considers the macro-level characteristics. Moreover, each unique implant’s pros and cons should also be evaluated regarding the needed esthetic demand, preferred loading time, and quality of bone.

While it is apparent that different modifications have a range of beneficial effects, it is essential to consider at what time point and in what conditions these effects occur. Certain modifications show significant results a very short time after implant placement, and equivalent results to controls a few weeks after placement. This suggests that particular modifications, such as creating a hydrophilic SLA implant, or UV photofunctionalization may be most beneficial in patients who require early loading protocols. Further studies with direct comparison among different surface modifications at various short- and long-term time frames are important to analyze to fully understand the indications, as well as the risks and benefits for each variation. Compared to the conventional drilling technique for implant site osteotomy, the osseodensification drilling protocol significantly increases implant insertion torque and bone to implant contact, which is beneficial for implant stability in poor density alveolar ridges [257]. However, all the tested implants in these studies are regular titanium implants. Investigating a proper combination of implant site osteotomy, implant material, and implant surface modification strategy has the great potential to improve the implant success rate, especially for the patients who have poor bone quality in the implant sites. In this constantly evolving field, future studies will continue to strive for a more evolved implant surface that achieves the precise combination between rapid and enhanced osseointegration and the limitation of biofilm formation.

## Figures and Tables

**Table 1:** The list of titanium implants’ surface modification approaches.

Modifying Approach	Category	Sub-Category	Technique/Resulting Surface	Notable Effect on Osseointegration/Biofilm Formation	Ref.
Shape	Physical	Macro	Tapered apex	Improves primary stability, favorable for immediate placement and immediate loading, superior in site with root proximity	[45,46]
Diameter and Length	Physical	Macro	Increased implant diameter	Increases ISQ, improves force distribution, reduces stress along implant length, elevates load-bearing capacity of prosthesis	[47,48]
Threads	Physical	Macro	V shape threads	Thread design that achieves most stability, least stress;	[48]
Machining	Physical	Micro	Polished surface with average roughness of 0.96 μm	Slightly promotes osteogenic cells to attach and deposit bone matrix	[32,49]
Grit-blasting	Physical	Micro	Roughened surface created by titanium dioxide particles of 25–50 um	Higher success rates compared to machined implants, more predictable long-term clinical outcomes	[50,51]
Acid-etching/Sandblasting	Physical	Micro	Macrostructure remodeled by large grit particles (250–500 μm), micro-irregularities created by HNO_3_/HF	Improves bioadhesion, accelerates osteoblast attachment and retention, facilitates osseointegration and bone apposition, increases bone anchorage; More severe colonization in vitro compared to machined and acid-etched implants	[50,52,53]
Laser Ablation	Physical	Nano	Complex, precise, high-resolution geometry generated	Induces significantly more bone-to-implant contact, larger torque removal values compared to machined surface, encourages attachment of connective tissue and bone; Lower biofilm formation compared to machined and grit-blasted surfaces and enamel	[54,55,56]
Nanocomposite	Physical	Nano	Various nanoparticles added through nanoparticle compaction, plasma spraying deposition, physical vapor deposition, and hot isostatic pressing	Improves biocompatibility and osseointegration; Reduces biofilm formation (*Staphylococcus aureus*)	[57,58,59]
Hydrophilic	Chemical		Hydroxylated (or hydrated), rinsed under nitrogen protection, and stored in isotonic saline solution	Encourages cell attachment and migration, promotes osteoblast differentiation/maturation, enhances neoangiogenesis, improves bone-to-implant contact, increases bone density, benefits earlier stages of osseointegration; Discourages hydrophobic bacterial attachment (*P. gingivalis, A. actinomycetemcomitans*, and *F. nucleatum*)	[60,61,62,63,64]
Discrete Crystalline Deposition	Chemical		Calcium phosphate particles of 20–100 nm compose 50% of surface	Better osteoconduction compared to cpTi/Ti6Al4V, increases bone-to-implant contact, beneficial for immediate loading; Reduction of bacterial adhesion (*A. actinomycetemcomitan, S. mutans*, and *S. sanguis*)	[65,66,67,68]
Anodic Oxidation/Anodization	Chemical		TiO_2_ layer (600–1000 nm) produced	Encourages gingival fibroblast deposition/adhesion/proliferation, and osteoblast adhesion, improves bone-to-implant contact, higher success rate versus machined implants during immediate loading; Reduction of bacterial adhesion by 1–2 logs	[32,50,69,70,71]
Fluoride Treatment	Chemical		Cathodic reduction reaction applies fluoride to surface	Stimulates undifferentiated osteoblasts/osteoprogenitor cells to proliferate/differentiate and have increased alkaline phosphatase activity, more firm bone-to-implant contact, greater removal torque; Facilitates the structural disruption and detachment of biofilm	[54,72,73,74,75,76]
Hydroxyapatite	Chemical		40–50 um HA layer created by plasma spraying	Beneficial where rapid bone-to-implant contact is needed; Antibacterial effects against *S. aureus* and *P. gingivalis*	[77,78]
UV Treatment/Photofunctionalization	Chemical		UVA (320–400 nm) and UVC (200–280 nm) waves alter hydrophilicity of TiO_2_	Enhances osteogenic cell attachment/proliferation and plasma protein adsorption, promotes bone formation in the early phase of osseointegration; Reduction in the attachment of *S. mutans, S. salivarius,* and *S. sanguis*	[54,79,80]
Atmospheric Pressure Plasma Processing	Chemical	Plasma	Hydrophilicity increased with piezo brush	Increased cell adhesion, alkaline phosphatase activity, and factors related to bone differentiation; Reduction in Gram-negative bacteria	[81,82]
Plasma Oxidation	Chemical	Plasma	Radio frequency plasma-enhanced chemical vapor deposition system achieves plasma oxidation	Increases removal torque and bone-to-implant contact	[83]
Calcium Chloride Treatment	Chemical		Hydrothermal treatment with calcium chloride (CaCl_2_)	Improves osseointegration and soft tissue seal, adsorption of laminin-332 and osteopontin and adhesion of osteoblasts, greater attachment of gingival epithelial-like cells and fibroblasts; No enhancement of bacterial adhesion	[19]
Platelet Rich Plasma	Biological		PRP with zoledronic acid applied to surface	Enhances initial bone apposition and primary healing	[84]
Extracellular Matrix (ECM)	Biological		Coating of collagen/chondroitin sulfate or collagen/sulfated hyaluronan	Enhances bone healing, increases bone formation and maturation compared to uncoated implants	[85,86]
RGD Peptide	Biological	Peptides	Specific amino acid sequence applied to surface	Improves bone-to-implant contact three months post-implantation, no effects on bone-to-implant contact, new bone fill, or removal torque values two weeks post-implantation	[78,87]
P15 Peptide	Biological	Peptides	Synthetic 15 amino acid peptide applied to surface	Promotes osseointegration, increases osteoblast and mesenchymal cell attachment/spreading, and osteogenic gene expression and differentiation	[88,89]
Strontium Incorporated Protein	Biological	Peptides	Magnetron sputtering/hydrothermal treatment coating method used	Improves bone-to-implant contact, bone formation, and biomechanical properties, enhances early adhesion, proliferation, and osteogenic differentiation of bone marrow stromal cells, increases expression of osteogenic related genes (i.e., BMP-2), and significant capability of new bone formation in vivo	[90,91,92,93]
Bactericidal Peptides	Biological	Peptides	GL13K and human beta defensins (HBDs) applied to surface	GL13K maintains cytocompatibility with adequate proliferation of osteoblasts/gingival fibroblasts. HBDs maintain the proliferation of osteoblasts and mesenchymal stem cells; GL13K has a bactericidal effect against *P. gingivalis.* HBDs exhibited broad-spectrum antibacterial functions	[94,95]
Sclerostin-Antibody	Biological	Peptides	Decreased sclerostin	Promotes osteoblast differentiation	[96]
Bone Morphogenic Protein (BMP)	Biological	Growth Factors	BMP-2 coating	Promotes bone regeneration, increases density of surrounding bone compared to acid-etched implants, improves bone-to-implant contact and new bone formation compared to anodized implants	[97,98,99]
Platelet-Derived Growth Factor (PDGF)	Biological	Growth Factors	PDGF coating	Enhances osseointegration, accelerates soft tissue healing around implant surface	[100,101]
Fibroblast Growth Factor (FGF)	Biological	Growth Factors	FGF-2 nanoparticle coating	Increases cell spreading and differentiation in vitro, increases osseointegration in rabbit tibia in vivo	[102]
Statins	Biological	Drugs	Simvastatin	Increases alkaline phosphatase activity, type I collagen synthesis, and osteocalcin release from pre-osteoblasts in vitro	[103]
Bisphosphonates	Biological	Drugs	Pamidronate disodium, ibandronate	Strengthens mechanical fixation; Increases bacterial adhesion and biofilm formation	[104,105]
Antimicrobials	Biological	Drugs	Tetracycline/Vancomycin	Tetracycline coating promoted cell proliferation and bone healing. Vancomycin coating boosted bone healing; Tetracycline killed contaminating microorganisms; Vancomycin inhibited colonization by *S. aureus*	[106,107]

**Table 2:** The list of zirconia implants’ surface modification approaches.

Modifying Approach	Category	Technique/Resulting Surface	Notable Effect on Osseointegration/ Biofilm Formation	Ref
Machined	Physical	Polished surface with average roughness of 0.96 μm	Decreased cell proliferation of osteoblasts, similar bone-to-implant contact compared to machined titanium; Significant decrease in biofilm thickness compared to machined titanium	[213,217,219]
Acid-etching/Sandblasting	Physical	Sandblasted with 250 mm sized particles	Enhances osteoblast cell adhesion in vitro; Grit-blasted with or without acid-etching significantly prohibits *S. sanguis* and *P. gingivalis* biofilm formation	[219,220]
Laser Modification	Physical	Increased surface free energy/wettability	Bone-to-implant contact and crestal resorption at one and three months comparable to SLA titanium implants, greater bone-to-implant contact and removal torque versus machined zirconia, increased cell proliferation, phosphatase activity, osteocalcin expression, and calcification	[221,222]
Coatings	Physical	Coating with silica, magnesium, nitrogen, carbon, and HA	Silica coating enhances HA formation and osteoblast proliferation. Magnesium coatings induce more cell proliferation and differentiation compared to pure zirconia-calcium phosphate coatings. HA-coating increases volume of new bone formation; Silica coating reduces bacterial adhesion. C:H:N layer harbors less initially adherent microorganisms	[223,224,225,226]
UV Treatment	Chemical	Surface free energy, wettability, and hydrophilicity enhanced by electron excitation	Enhances attachment, proliferation, and differentiation of osteoblasts, faster osseointegration, greater bone volume and bone-to-implant contact	[213,227,228]

**Table 3:** The list of PEEK implants’ surface modification approaches.

Modifying Approach	Category	Technique/Resulting Surface	Notable Effect on Osseointegration/ Biofilm Formation	Ref
Roughening	Physical	Acid-etched or sandblasted	Improve osseointegration	[239,240]
Reinforcing	Physical	Reinforced with different materials (i.e., glass/carbon), fiber lengths and orientations changed	Carbon-fiber-reinforced PEEK (CFR-PEEK) improves bone-to-implant contact, but increases stress concentrations compared to titanium implants, exhibits black coloration	[206,238,241]
Creation of Nanocomposites	Physical	HA or titanium nanoparticles applied through melt-blending/nano-scale fluorohydroxyapatite	PEEK/CF/n-HA promotes osteogenesis and encourages cell growth, cell attachment, and proliferation. PEEK/nano-HAF increases bone cell proliferation; PEEK/nano-HAF possesses antibacterial properties against *S. mutans*	[208,242,243]
Nitration, Sulfonation, Amination	Chemical	Chemical groups introduced to surface	Enhances biocompatibility, achieves early osseointegration; Sulfonation treatment used to incorporate lactam-based antibiofilm establishes a surface more resistant to bacterial contamination (*S. aureus* and *Escherichia coli)*	[206,208]
UV Treatment	Chemical	Increased hydrophilicity/wettability	Favorable for early attachment of soft tissue and cell proliferation, attachment of fibroblasts	[206,242,244]
Plasma	Chemical	Gas plasma (exposure to low-power plasma gases such as water vapor, ammonia, oxygen/argon, and nitrogen) introduces several functional groups to the surface. Plasma immersion ion implantation accelerates and deposits ions onto the surface, creating a thin film of various particles	Gas plasma treatment accelerates mesenchymal cell proliferation for bone formation. Nitrogen-plasma-treatment increase torque removal values and bone formation, comparable to titanium implants; Fluorinated PEEK created through plasma immersion ion implantation improves cell adhesion, cell spreading, proliferation, and alkaline phosphatase activity compared to pure PEEK; Fluorinated PEEK shows bacteriostatic properties against *P. gingivalis*	[242,245,246,247]
Coating Techniques	Chemical	Spin coating (thin layer of nanohydroxyapatite created by spinning implant at high speed and then heat-treating). Electron beam deposition (thin titanium coating deposited)	Spin-coating with nanohydroxyapatite leads to higher removal torque value; Electron beam deposition of thin titanium coating increases cellular adhesion, significantly greater bone-to-implant contact than uncoated PEEK implants	[242,248,249,250]
Antibacterial Modification	Chemical	Silver-nanoparticles	Increases success rate and reduces peri-implantitis; Titanium pentoxide (Ti_3_O_5_) combined with 12.5% silver applied on PEEK surfaces effective against *S. aureus*	[251,252]
